# Facile fabrication of antibacterial and antiviral perhydrolase-polydopamine composite coatings

**DOI:** 10.1038/s41598-021-91925-6

**Published:** 2021-06-14

**Authors:** Li-Sheng Wang, Shirley Xu, Sneha Gopal, Eunsol Kim, Domyoung Kim, Matthew Brier, Kusum Solanki, Jonathan S. Dordick

**Affiliations:** 1 Department of Chemical and Biological Engineering and Center for Biotechnology & Interdisciplinary Studies, Rensselaer Polytechnic Institute, 110 8th Street, Troy, 12180 USA; 2 Department of Biological Sciences, Rensselaer Polytechnic Institute, 110 8th Street, Troy, NY 12180 USA; 3 Department of Biomedical Engineering, Rensselaer Polytechnic Institute, 110 8th Street, Troy, NY 12180 USA

**Keywords:** Antimicrobials, Antiviral agents, Biocatalysis, Immobilized enzymes

## Abstract

In situ generation of antibacterial and antiviral agents by harnessing the catalytic activity of enzymes on surfaces provides an effective eco-friendly approach for disinfection. The perhydrolase (AcT) from *Mycobacterium smegmatis* catalyzes the perhydrolysis of acetate esters to generate the potent disinfectant, peracetic acid (PAA). In the presence of AcT and its two substrates, propylene glycol diacetate and H_2_O_2_, sufficient and continuous PAA is generated over an extended time to kill a wide range of bacteria with the enzyme dissolved in aqueous buffer. For extended self-disinfection, however, active and stable AcT bound onto or incorporated into a surface coating is necessary. In the current study, an active, stable and reusable AcT-based coating was developed by incorporating AcT into a polydopamine (PDA) matrix in a single step, thereby forming a biocatalytic composite onto a variety of surfaces. The resulting AcT-PDA composite coatings on glass, metal and epoxy surfaces yielded up to 7-log reduction of Gram-positive and Gram-negative bacteria when in contact with the biocatalytic coating. This composite coating also possessed potent antiviral activity, and dramatically reduced the infectivity of a SARS-CoV-2 pseudovirus within minutes. The single-step approach enables rapid and facile fabrication of enzyme-based disinfectant composite coatings with high activity and stability, which enables reuse following surface washing. As a result, this enzyme-polymer composite technique may serve as a general strategy for preparing antibacterial and antiviral surfaces for applications in health care and common infrastructure safety, such as in schools, the workplace, transportation, etc.

## Introduction

Everything we touch in our daily lives is inhabited by microorganisms, including Gram-positive and -negative bacteria and viruses that have the potential to transmit disease, from colds to more serious illnesses, as well as leading to widespread epidemics and pandemics. Even in our hospitals and clinics, germ-bearing surfaces can prove deadly, particularly due to the overuse of antibiotics leading to antibiotic-resistant strains^1^. In fact, one in 31 hospitalized patients contract a nosocomial infection according to the U.S. Center for Disease Control and Prevention (CDC)^2^. These infections result from bacterial contamination of material surfaces such as bedrails and linens, intravenous needles, catheters and implants, and are major concerns for healthcare^3^. Bacterial contaminations also pose food safety concerns in this age of worldwide transport as food-borne illnesses sicken millions and kill thousands of people each year^4^. Furthermore, in many bacterial surface contaminations, a particular challenge arises from biofilm formation where adherent bacteria proliferate and produce extracellular matrices that increase their resistance to disinfectants and antibiotics^5–7^.

Viral contaminations and infections represent another source of public health concern^8,9^, as there are few antiviral therapeutics, and unchecked viral transmission can lead to epidemics and pandemics. The most recent and tragic example is COVID-19 caused by the SARS-CoV-2 beta-coronavirus^10–12^, which as of November 2020 has infected nearly 60 million people worldwide leading to nearly 1.4 million deaths^13^. Even in the biomanufacturing sector, microbial and viral contamination of bioreactors causes significant loss of revenue and detrimental impacts on drug product safety and cell culture productivity^14,15^. As a result, development of active antimicrobial coatings is crucial for infrastructure, healthcare, biomanufacturing, and the food industry.

Various chemical disinfectants, such as bleach and phenols, are currently used for surface decontamination. Bleach, while highly effective, is also toxic and corrosive to many surfaces^6^. Phenols are less corrosive; however, they are stable and are considered a persistent bio-accumulative toxin by the U.S. Environmental Protection Agency (EPA)^7^. In addition, these and other chemical disinfectants often pose health risks and environmental burdens as they need to be applied continuously at high concentrations^16^. Antibiotic use, while once common, is under pressure due to the increasing prevalence of resistant strains^17^. As an alternative to chemical decontaminants and antibiotics, enzymes have been used to generate disinfectant species in situ. For example, laccases, multicopper oxidases, catalyze the oxidation of iodide into molecular iodine that is highly toxic to microbes^18^ and viruses^19^, and haloperoxidases, such as chloro- and bromoperoxidase, generate antimicrobial hypohalous acids in the presence of chloride or bromide and hydrogen peroxide (H_2_O_2_)^20^. With respect to the current work, perhydrolases in the presence of H_2_O_2_ and an acetate donor, such as propylene glycol diacetate (PGD), generate the highly antimicrobial peracetic acid (PAA)^21,22^.

Such enzyme-based disinfectants have been tested in liquid formulations; however, to reduce enzyme degradation and the recurring need for reapplication, as is currently done with chemical disinfectants and antibiotics, these enzymes would be most effectively utilized by embedding them into surface coatings^23^. Creating such enzymatic coatings would yield self-decontaminating surfaces that, by maintaining enzyme activity and stability, would have sufficient lifetimes for long-term use. Due to the peracid products of perhydrolases, such as PAA that would be slowly and controllably generated, immobilization strategies are particularly of interest.

PAA is a strong oxidant with broad spectrum activity against pathogens and, for this reason, has become a common sterilant in healthcare and other industries^24^, including being approved for environmental use as a sporicidal agent^25^ and as a pesticide^26^, and approved for use in the food industry^27^. However, chemical synthesis of PAA involves sulfuric acid catalysis, leading to highly corrosive byproducts^28^, and the transport of PAA is challenging due to its high reactivity^29^. These drawbacks make controlled in situ enzymatic synthesis of PAA attractive^30^. To this end, Matthews et al. determined that perhydrolase S54V (denoted as AcT, MW 180 kDa) from *Mycobacterium smegmatis* can use various aliphatic esters as acyl donor substrates and H_2_O_2_ as nucleophile to generate peracids with far higher perhydrolysis activity than hydrolysis activity^31^, the latter more typical of lipases and esterases^22^. In previous studies, we immobilized AcT onto multiwalled carbon nanotubes (MWNTs) with PEG spacers and showed retention of approximately one-quarter of the enzyme’s native activity in aqueous solution^21^. To fabricate antimicrobial coatings, we further incorporated the AcT-PEG-MWNTs into latex-based paint formulations and demonstrated their sporicidal and antiviral activity^23^. Nonetheless, this approach required multiple steps, including stabilization onto a high aspect ratio nanomaterial.

As an alternative approach, polydopamine (PDA) is a biomimetic polymer that can be deposited as a thin film onto virtually any surface^32^. Under slightly alkaline and oxidative conditions, dopamine polymerizes and gives rise to quinone, indole, and catecholamine moieties^29^. The presence of the amine and quinone groups facilitates crosslinking and leads to a heteropolymer that forms a number of supramolecular interactions, including hydrogen bonding, pi-stacking, and pi-cation interactions^33–35^. This gives PDA structural similarity to the mussel (*Mytilus edulis*) adhesive foot protein-5that is rich in l-3,4-dihydroxyphenylalanine and l-lysine, which are responsible for its strong adhesive properties^36^. Furthermore, the quinone and catechol groups on the PDA readily react with thiol and amino functional groups, including surface moieties on proteins and nanoparticles^37^, allowing for PDA-coated surfaces to be modified and functionalized. For example, Fan et al*.* grafted zwitterionic antifouling sulfobetaine moieties onto PDA-coated catheters to reduce bacterial adhesion^38^ Similarly, Liu et al*.* incorporated silver nanoparticles into wound dressings coated with PDA to introduce antibacterial activity^39^. These adhesive properties have also led to the development of PDA-based wet surface adhesives^33^.

PDA’s unique adhesive properties have already been exploited for enzyme immobilization to prepare biocatalytic membranes^40^, microreactors^41^, and functionalized nanoparticles^42^. However, to date, the majority of PDA-based enzyme immobilization studies have used a multi-step method in which the enzyme of interest is attached to the surface after the PDA coating is formed^43^. Thus, the enzyme is simply surface immobilized, a restriction in the synthesis method that reduces enzyme loading and subjects an enzyme to environmental factors, such as proteases and surface fouling that can lead to deactivation. An alternative approach would be a one-step, in situ immobilization of enzyme during the PDA deposition process, which would incorporate the biocatalyst throughout the coating material^44^.

In the current work, we have developed a versatile immobilization strategy for the fabrication of enzymatic decontamination surface coatings. This approach employed a single-step PDA self-polymerization with in situ enzyme incorporation during surface deposition. We hypothesized that the in situ enzyme-polymer composite strategy would yield higher AcT loadings in the coatings as compared to the more traditional surface functionalization approach, and provide a method that would be expected to incorporate AcT onto virtually any surface. This hypothesis was tested by evaluating broad-spectrum antimicrobial activity of AcT-coated surfaces on polystyrene (PS), epoxy, glass, and stainless steel surfaces, as well as antiviral activity against a common lentivirus and a pseudovirus to SARS-CoV-2. As a result of this strategy, we posit that a PDA-based composite enables the efficient incorporation of AcT onto myriad materials in diverse environments to create self-decontaminating, antibacterial and antiviral surfaces.

## Materials and methods

### Materials

Propylene glycol diacetate (PGD), H_2_O_2_ solution (30%), kanamycin, isopropyl-β-d-thiogalactoside (IPTG), peracetic acid (PAA), and dopamine were purchased from Sigma (St. Louis, MO). 2,2′-Azino-bis(3-ethylbenzothiazoline-6-sulfonic acid)-diammonium salt (ABTS) was purchased from Pierce (Rockford, IL). Luria–Bertani (LB) medium was purchased from Becton Dickinson (Franklin Lakes, NJ). Trypticase soy broth (TSB) was purchased from MP Biomedicals (Irvine, CA). Stainless steel coupons were obtained from Metal Fabrication, Inc., (Albany, NY) and epoxy flooring coupons were obtained from CTI, Inc. (Ravena, NY).

### Protein expression and purification

The *Act* gene was synthesized by GenScript (Piscataway, NJ). The amplified *Act* was subcloned into a pET28a plasmid between NcoI and BamHI restriction sites with a His_8_ tag and thrombin cleavage site (LVPRGS) at the N-terminus. Plasmid constructs were transformed into *Escherichia coli* strain BL21 (DE3). The transformed *E. coli* cells were incubated in LB medium containing kanamycin (50 μg mL^−1^) at 37 °C until they reached an optical density at 600 nm (OD_600_) of 0.4 to 0.6. Expression of recombinant AcT protein was induced by adding 1 mM IPTG. After 16 h, the cell cultures were collected, centrifuged, and cell pellets were resuspended in phosphate-buffered saline (PBS) at pH 7.4. To harvest the AcT, cell suspensions were sonicated using a Sonics Vibra (Newtown, CT) cell for 10 min at 60% amplitude with 3 s pulses and then centrifuged at 10,000 rpm for 15 min. The His_8_-tagged AcT was purified from the supernatant via nickel nitrilotriacetic acid affinity chromatography (Gold Biotechnology, St. Louis, MO). Eluents were dialyzed against PBS using 12–14 kDa molecular weight cut-off dialysis tubing and then filtered through a 0.2-μm pore size polyethersulfone membrane (Millipore, Burlington, MA). Sample purity was determined using sodium dodecyl sulfate–polyacrylamide gel electrophoresis (SDS-PAGE) where gels were stained with shaking for 1 h at room temperature using Gelcode™ Blue Safe Protein stain and then destained overnight in water. Protein concentrations were determined using the bicinchoninic acid (BCA) assay (ThermoFisher, Waltham, MA).

### Fabrication of AcT-PDA composite coatings

For the 2-step PDA coating synthesis method, the coating was prepared using 100 μL of 1 mg mL^−1^ dopamine solution (in PBS, pH 7.4) in wells of polystyrene 96-well plates and incubating overnight at room temperature. The PDA solution was removed after overnight incubation and the plates were washed with 200 µL PBS prior to adding enzyme. Then, 1–5 μg AcT was added in 100 μL PBS and the enzyme was allowed to react with the preformed PDA for 12 h. This resulted in 1–5 μg AcT per approximately 100 μg PDA coating. The catalytic activity of the AcT bound to PDA was determined by measuring PAA generated over time in the presence of a 200 μL solution containing 100 mM PGD and up to 200 mM H_2_O_2_. For fabrication of single-step AcT-PDA composite coatings, an immersion procedure was used in which a dopamine/AcT solution in PBS (pH 7.4) was prepared with 1 mg mL^−1^ dopamine and 10–50 μg mL^−1^ AcT. For typical experiments, 100 μL of dopamine/AcT solution were added to wells in a 96-microwell plate and incubated overnight. After incubation, the supernatant was removed and the surface was washed three times with PBS. The resulting coatings were stored in 200 μL PBS to avoid drying.

Quantification of AcT within the PDA composites was performed using SDS-PAGE analysis. Following overnight incubation, the remaining dopamine/AcT solutions were collected and run on SDS-PAGE. A standard curve of band intensity as a function of protein concentration was obtained by image analysis using ImageJ (National Institutes of Health). The amount of AcT incorporated into the PDA composite was calculated by subtracting the free AcT left in solution from the original amount pre-incubation.

For atomic force microscopy (AFM) characterization, silicon wafers (UniversityWafer, South Boston, MA) were cut into 1 cm squares and placed in wells of a 24-microwell plate followed by 500 μL of dopamine/AcT solution. After overnight incubation, the coated silicon wafers were removed from the solution, washed three times with PBS, and then dried under nitrogen gas before AFM measurements. AFM measurements were performed using an Asylum MFP-3D AFM (Oxford Instruments, Concord, MA) under tapping mode.

Similarly, for x-ray photoelectron spectroscopy (XPS) characterization, 1-cm^2^ silicon wafers were coated using 500 μL of dopamine/AcT solution in the wells of a 24-microwell plate, incubated overnight, washed three times with PBS, and then dried under nitrogen gas. XPS measurements were performed using a PHI 5000 Versaprobe XPS (Physical Electronics, Chanhassen, MN) with a monochromated Al x-ray source. XPS survey scans were conducted on samples from 1000 to 0 eV at 1 eV increments and were run five times to amply signal and reduce noise. Scans of key regions, including the C1s (298 to 278 eV at 0.1 eV increments), N1s (411 to 391 eV at 0.1 eV increments), and O1s (543 to 523 eV at 0.1 eV increments), were also conducted to identify elemental composition and bonding interactions; these regions were each scanned 50 times to enhance the signal-to-noise ratio.

### AcT activity assays

AcT activity in free solution and in AcT-PDA composites was determined by measuring PAA generation. PGD/H_2_O_2_ solutions in PBS (pH 7) were prepared with final concentrations of up to 100 mM PGD and 10 mM H_2_O_2_. The resulting PGD/H_2_O_2_ solutions (200 μL) were added into wells of a 96-microwell plate containing either 20 μg mL^−1^ free AcT or the AcT-PDA composite coatings prepared with different concentrations of AcT. The reactions were performed for up to 30 min at room temperature. PAA quantification was performed by diluting the reaction solution five to tenfold in PBS and then mixing 20 μL of this diluted solution with 180 μL of ABTS assay reagent (10 mM ABTS and 50 μM potassium iodide in 125 mM potassium citrate buffer, pH 5.0). The mixture was then incubated at room temperature for 10 min after which the absorbance at 420 nm was measured. PAA concentration was calculated by using a standard curve prepared at different concentrations of PAA.

Steady-state kinetics were determined in by varying the concentration of PGD from 3 to 100 mM while maintaining the H_2_O_2_ concentration at 200 mM (all dissolved in 200 μL PBS) at an AcT loading of 20 μg mg^−1^ PDA. Initial reaction rates for PAA generation were obtained by removing 10 μL reaction aliquots every minute for 5 min, diluting the aliquots two to tenfold with PBS, mixing the diluted samples with the ABTS assay reagent for 10 min, and then measuring the absorbance at 420 nm.

### Stability and reusability tests

The thermal stability of AcT-PDA composite coatings was determined using coatings prepared with 50 μg mL^−1^ AcT, 100 mM PGD and 10 mM H_2_O_2_ at 25–70 °C. The amount of PAA generated was determined after a 10 min reaction using the ABTS assay. The reusability of AcT-PDA composite coatings was assessed by cyclic PAA generation at 25 °C. Composites were tested for PAA production in the presence of 100 mM PGD and 10 mM H_2_O_2_ for 10 min then placed in PBS for 10 min before washing three times with PBS and repeating the process. The PAA generated during each 10 min cycle was quantified using the ABTS assay.

### AcT-PDA antimicrobial activity

The bacteria tested were obtained from ATCC (Manassas, VA). The antimicrobial activity of the AcT-PDA composite coatings was determined by adding 200 μL of a microbial suspension containing 10^7^ colony forming units (CFU) mL^−1^ (resulting in a challenge of 2 × 10^6^ CFU) to PDA (control) or AcT-PDA coated polystyrene wells of 96-microwell plates and incubated for 30 min in the presence of 100 mM PGD and 10 mM H_2_O_2_. After incubation, aliquots from the mixtures were collected, diluted (ca. tenfold), and plated on TSB agar (0.75% w/v agar). The number of surviving bacteria, represented as CFU, were counted after overnight incubation. The activity of AcT-PDA composite coatings was calculated in comparison to the CFUs of non-AcT containing coating controls that were collected and plated in the same manner as described above, although the dilution factor was increased accordingly. For reusability, the AcT-PDA coatings were washed three times with PBS after each reaction cycle, following by plating as described above.

### Fabrication and antimicrobial testing of AcT-PDA coatings on different surfaces

Epoxy floor material, stainless steel, and glass coupons were placed in dopamine/AcT solution (1 mg mL^−1^ and 50 μg mL^−1^, respectively) and incubated overnight. The coupons were washed three times with PBS and placed individually into sterile petri dishes. The antimicrobial activity of bare and coated coupons was determined by adding 200 μL of a microbial suspension containing 10^7^ CFU mL^−1^ onto the coupons and incubating for 30 min in the presence of 10 mM PGD and 1 mM H_2_O_2_. After incubation, 20 mL of TSB agar was poured onto the coupons and the surviving bacteria were observed after 24 h.

### Antiviral activity of AcT-PDA coatings

Antiviral activity of AcT-PDA coatings was tested on two different viral strains; a VSV-g pseudotyped lentivirus and a SARS-CoV-2 spike pseudotyped lentivirus, both carrying an *Egfp* reporter gene. To produce both viruses, HEK293T cells were seeded in two T-175 flasks and cultured in Dulbecco’s Modified Eagle’s Medium (DMEM) supplemented with 10% Fetal Bovine Serum (FBS). When cells were 70–80% confluent, they were transfected using Lipofectamine 2000 (Thermo Fisher Scientific, Waltham, MA) with 26 µg of psPAX2 per flask (a gift from Didier Trono, Addgene plasmid # 13360), 26 µg of pLV-EGFP per flask (a gift from Pantelis Tsoulfas, Addgene plasmid # 36083) along with either 6.89 µg pMD2.g per flask (a gift from Didier Trono, Addgene plasmid #12259) for VSV-g pseudotyped lentivirus production or 8.7 µg per flask of pHDM-SARS-COV2-S (BEI Resources #NR52514) for SARS-CoV-2 spike pseudotyped lentivirus production. A medium exchange was performed 24 h post-transfection at which point 5 mM of sodium butyrate was added to the medium. The supernatant containing either the VSV-g pseudotyped lentivirus or SARS-CoV-2 spike pseudotyped lentivirus was harvested at both 48 h and 72 h after transfection and pooled together. The pooled supernatants were concentrated using Lenti-X-Concentrator (Takara Bio, Shiga, Japan) according to manufacturer’s instructions and resuspended in DMEM. The pseudovirus production protocol was modified based on a previously published protocol^45^. The concentrated viruses were then titered using HEK293T (for VSV-g pseudotyped lentivirus) or HEK293T-Ace2 stable cell line (for SARS-CoV-2 pseudotyped lentivirus) and used for anti-viral assays.

To prepare the AcT-PDA coatings, a 100 μL solution containing 1 mg mL^−1^ of dopamine (in PBS) and 10 μg mL^−1^ of AcT was prepared and placed into wells of a 96-well plate. After overnight incubation, the wells were washed three times with 200 μL PBS followed by addition of 100 μL of PBS containing the lentivirus or pseudovirus, 5 mM PGD and 2.5 mM H_2_O_2_. The resulting mixture was incubated at room temperature. At both 0 min and 5 min time points, 100 μL of a quenching solution containing 100 U mL^−1^ catalase and 0.05% thiosulfate was added to wells to decompose both residual H_2_O_2_ and the PAA produced to avoid killing the mammalian cells in the subsequent viral titer assay. Following quenching of the reaction, the virus was diluted to obtain a multiplicity of infection (MOI) of 1 for lentivirus and 0.2 for SARS-CoV-2 pseudovirus and introduced to HEK293T cells and HEK293T-Ace2 stable cell line, respectively, in the presence of 8 µg mL^−1^ polybrene. A lower MOI for SARS-CoV-2 pseudovirus was used because pseudovirus production was not as efficient as lentivirus production and generated lower viral titers. A medium exchange was carried out 24 h post-infection and the plates were imaged 72 h post-infection. The cells were stained with Hoechst 33342 dye at a concentration of 5 µg mL^−1^ to label nuclei and were imaged using Cellomics ArrayScan XTI High Content Analysis (HCA) Reader (Thermo Fisher Scientific, Waltham, MA). The percentage of infected cells was calculated using the Target Activation BioApplication on the HCA reader by identifying the number of GFP positive cells in each well divided by the total number of cells in that well (as identified by the nuclear stain).

## Results and discussion

**Figure 1:**
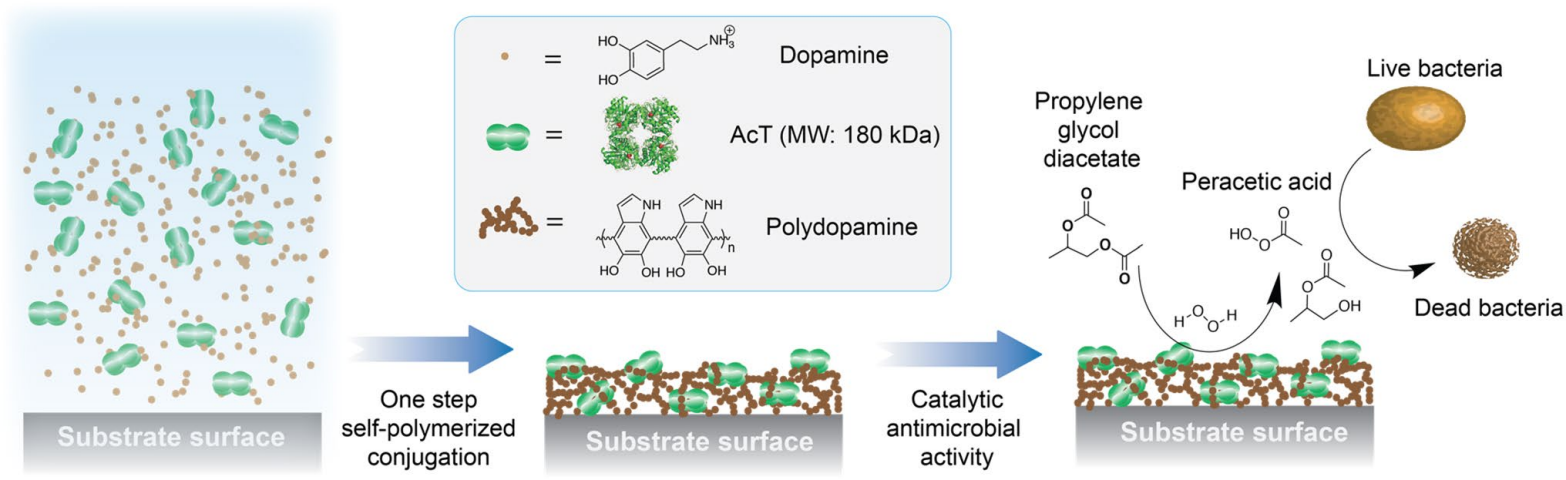
Schematic illustration of single-step fabrication of AcT-polydopamine composite coatings. Incorporation of AcT into polydopamine on a flat surface, via entrapment and/or covalent reaction with the polymer matrix, leads to a stable enzyme-polymer composite coating. The enzymatic reaction of AcT with propylene glycol diacetate and hydrogen peroxide to give peracetic acid is shown, leading to antimicrobial activity.

Dopamine serves as a simple, biological monomer that can undergo self-polymerization to PDA while retaining a number of functional groups that are ideal for biomolecule and surface attachment. Conventional use of PDA as a functionalized coating involves the formation of the PDA prior to attachment of surface species, including proteins^46^, although such an approach is only required for situations where the active surface components must be in direct contact with a target. However, a porous PDA with enzymes embedded and covalently attached within the polymeric matrix can serve as a catalytically active material, particularly if the enzyme substrates are small and readily diffusible through the coating matrix. This requires the addition of an enzyme to a dopamine solution followed by a single-step polymerization. In the current work, we generated AcT-integrated PDA composites as depicted in Fig. 1. Under slightly alkaline conditions and in the presence of air, dopamine oxidizes to PDA and, in the process, covalently binds to the free amino groups on AcT, thereby embedding it within the polymer network. This single-step in situ immobilization method results in AcT molecules embedded within a multilayered three-dimensional PDA structure.

### Fabrication and biocatalytic characterization of AcT-PDA composites

We initially examined AcT bound to preformed PDA using the more conventional 2-step process and resulting in surface attachment of AcT layer. The surface-attached AcT had relatively low activity; 20 μg AcT added to approximately 100 μg pre-formed PDA coating resulted in ~ 1.0 mM PAA in 30 min using 100 mM PGD and 10 mM H_2_O_2_ (Supplementary Fig. 1c). Furthermore, the activity of PDA surface-bound AcT minimally increased as a function of enzyme concentration, indicating that only a small fraction of the AcT remained bound to the surface PDA moieties. Indeed, as the concentration of AcT in solution increased above 30–40 μg mg^−1^ PDA, the bound AcT dropped (Supplementary Fig. 1c), such that at 50 μg mg^−1^ PDA, less than 15% of the added AcT remained unbound. This may be a result of restricted surface area or protein–protein interactions on the surface of the PDA, and subsequent removal from the surface due to pre-analysis washing.

**Figure 2:**
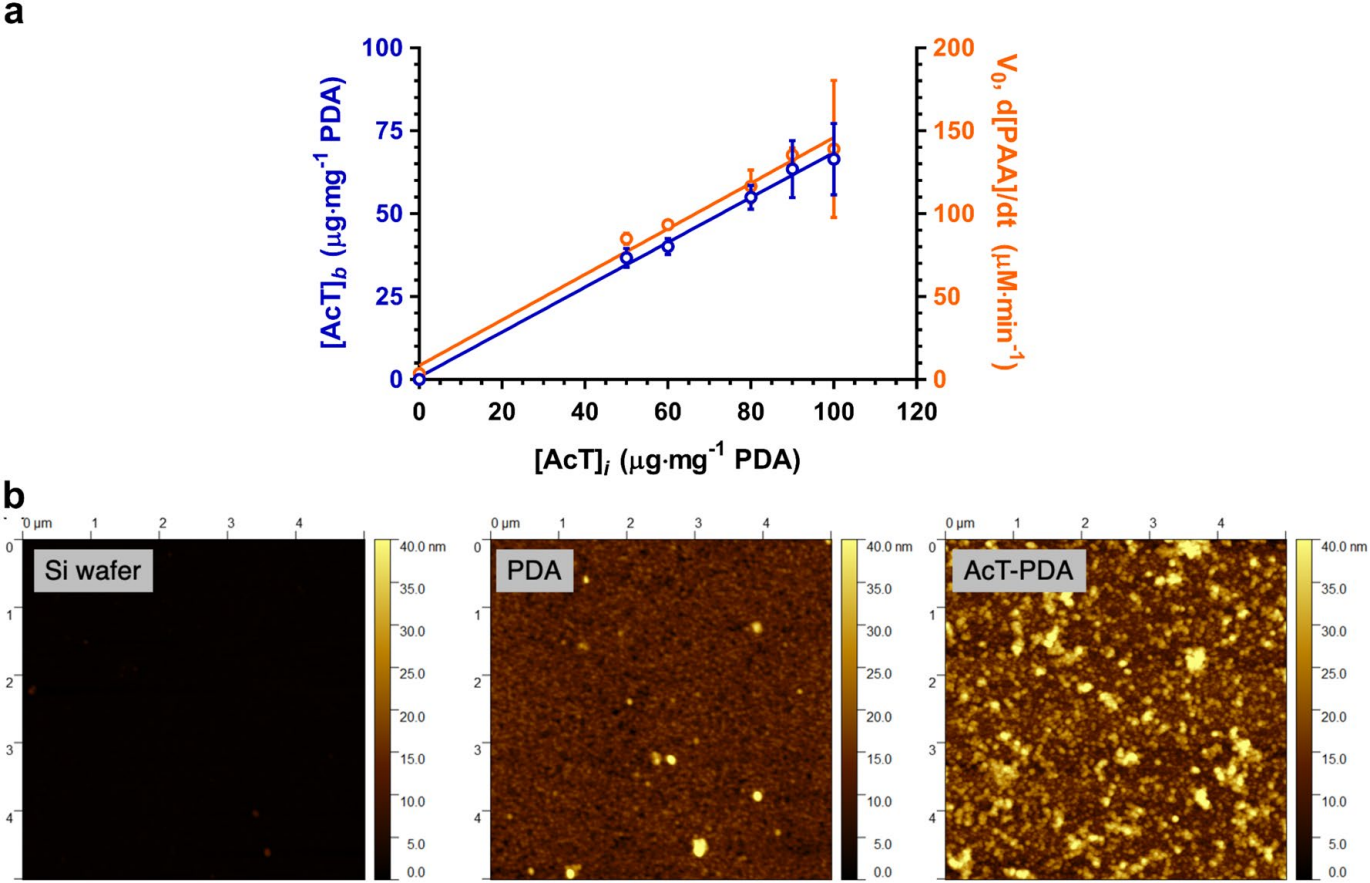
(**a**) Quantification of the 1-step incorporated AcT in the AcT-PDA coatings determined by SDS-PAGE analysis of the unbound AcT fraction (left y-axis) and catalytic activity of 1-step AcT-PDA coatings as a function of originally loaded AcT (right y-axis). For 1-step approach, 100 μL of 50–100 μg mL^−1^ AcT solution were mixed with 1 mg mL^−1^ dopamine solution in a 96-well plate overnight. The PAA was generated by adding 200 μL of 100 mM PGD and 10 mM H_2_O_2_ to the AcT coated plates, and incubated for 10 min. (**b**) AFM images showing the topographic changes from Si wafer substrate to PDA coating to the final AcT-PDA composite that reveals extent of AcT incorporation into the PDA coating.

The single-step AcT-PDA composite, on the other hand, resulted in anywhere from 70–80% of the added AcT being incorporated into the AcT-PDA composite, as determined by residual protein measured via SDS-PAGE analysis (Fig. 2a) to give an enzyme density of up to 20 μg per mg PDA (2 μg 100 μg^−1^ PDA per well). The catalytic activity of the AcT incorporated into the PDA composite was notably higher with up to ~ 3.3 mM PAA generated in 30 min (Figure S2), more than triple that of the surface-attached approach and approx. 50% of that produced by free enzyme under the same conditions (data not shown). The activity of the AcT-PDA composite coating was linearly dependent on enzyme concentration with a slope of approximately 1.4 (Fig. 2a).

AFM was used to assess the morphology and topography of the PDA-based coatings. In the absence of AcT, a thin layer of homogenous PDA coating was observed after overnight incubation (Fig. 2b). The nanoscale granular coating structure is consistent with results in the literature^47^, indicating that PDA-based coatings were formed using the simple immersion procedure. When AcT was added during the polymerization process, the resulting AcT-PDA composite coatings showed greater surface roughness with larger granules as compared to the pure PDA coatings. This suggests that the enzyme molecules crosslinked with the PDA to form AcT-enriched “islands” within a more homogeneous PDA background. These islands are roughly 25–35 nm in height with diameters ranging from 100–500 nm, suggesting nanoscale aggregates of AcT within the PDA. Despite these aggregates, however, the enzyme retained high activity. As a result of these experiments, we focused our attention solely on the single-step procedure to develop AcT-PDA composite materials. XPS analysis reveals the presence of AcT in the PDA polymer (Supplementary Fig. 2). Specifically, the increased intensity at 288 eV in C1s binding energy suggests an increased fraction of C=O in AcT-PDA as compared to PDA only, which indicates the formation of protein-PDA complexes.

### Reaction kinetics of AcT-PDA composite coatings

**Figure 3:**
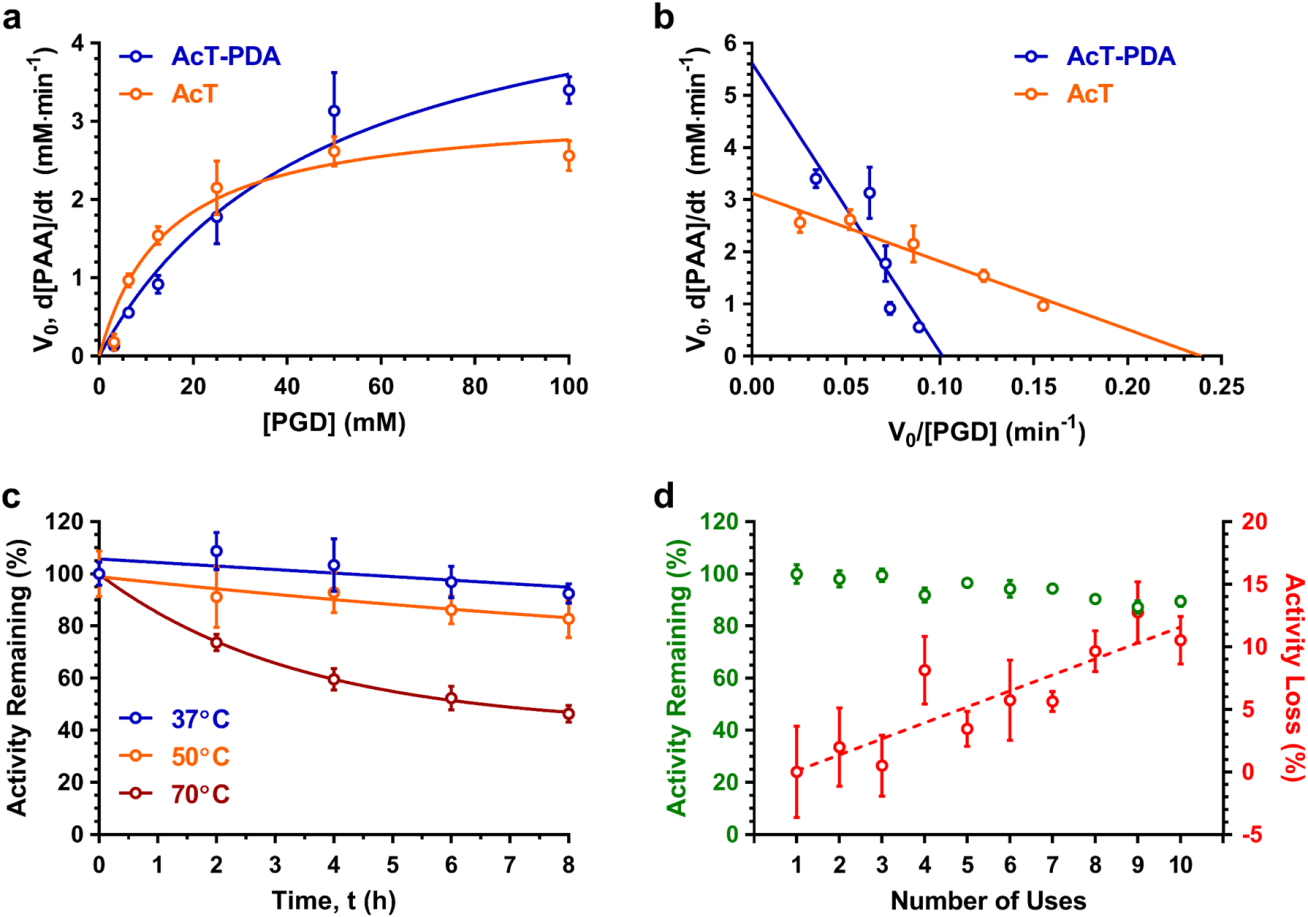
Reaction kinetics, thermal stability, and reusability of AcT-PDA composites. AcT-PDA composites (blue) loaded using an initial AcT concentrations, [AcT]_*i*_, of 20 μg AcT mg^−1^ PDA were compared to free AcT (at corresponding 20 μg AcT mL^−1^ reaction, orange) for AcT activity. Curves for reaction kinetics were fitted based on (**a**) the Michaelis–Menten equation and (**b**) an Eadie-Hofstee plot fit. (**c**) Residual activity of AcT-PDA composites ([AcT]_*i*_ = 50 μg AcT mg^−1^ PDA) after incubating at 37, 50, and 70 °C for up to 8 h. (**d**) Residual activity (green) of AcT-PDA composites ([AcT]_*i*_ = 50 μg AcT mg^−1^ PDA) as a function of number of uses at 25 °C. Activity loss (red) shown on expanded right axis to gradual decay in activity. All tests of coating activity were performed with *n* = 3 replicates. All data shown as mean ± standard deviation; error bars not plotted for some points that have very small standard deviations.

To gain additional insight into the nature of AcT-PDA composite coatings, we examined the steady-state kinetic profiles of both the composite and free AcT. Keeping the H_2_O_2_ concentration constant (200 mM) while varying the PGD concentration (3–100 mM), both free and immobilized AcT (2 and 10 μg mL^−1^, respectively) followed apparent Michaelis–Menten steady-state kinetics (Fig. 3a). It should be noted that AcT is stable to high H_2_O_2_ concentrations^23^. Based on Eadie-Hofstee plots (Fig. 3b), free enzyme had (*k*_cat_)_app_ and *K*_m_ values of 4.7 × 10^3^ s^−1^ and 13 mM, respectively, which resulted in a (k_cat_/K_m_)_app_ of 3.7 × 10^5^ M^−1^ s^−1^. Comparatively, for the AcT-PDA composites the (*k*_cat_)_app_ and K_m_ values were 1.7 × 10^3^ s^−1^ and 55 mM, respectively, thus having a (k_cat_/K_m_)_app_ of 3.1 × 10^4^ M^−1^ s^−1^. The apparent values for k_cat_ and k_cat_/K_m_ were used due to the two-substrate reaction and varying only the PGD. Thus, the AcT-PDA composite possessed ~ 40% of the catalytic turnover as that of the free enzyme, and this was approximately 10% higher than that for previously reported AcT-PEG-MWNTs^23^. The ~ fourfold higher *K*_m_ value for the AcT-PDA composite compared to the free enzyme is not surprising and suggests the presence of some diffusional limitations that restrict the rate of substrate access to the AcT embedded in the PDA coating.

To address potential substrate diffusion limitations, we calculated the Thiele modulus for a slab geometry^48^ based on Eq. (1);

1 $$\phi ={X}_{p}\sqrt{\frac{{k}_{cat}[{E}_{o}]}{{K}_{m}{D}_{eff}}}$$

where *X*_p_ represents the slab thickness, E_o_ represents the enzyme concentration per reaction volume (200 μL), and D_eff_ represents the effective diffusivity, which is assumed to be equivalent to small molecule bulk diffusion (10^–6^ cm^2^ s^−1^), as the PDA matrix was assumed to be highly porous. The slab had a thickness of 0.26 cm based on 100 μg PDA per well in a standard 96-well plate geometry. Based on the value of (k_cat_/K_m_)_app_ and an enzyme concentration of 10 μg mL^−1^, the value of *ϕ* = 10.7. The effectiveness factor, η, was calculated based on Eq. (2) to be 0.25. This value suggests that the AcT-PDA composite possesses moderate substrate diffusional restrictions. However, the reduced substrate diffusion indicated that the intrinsic catalytic efficiency of the AcT-PDA composite was roughly threefold lower than the free enzyme in solution (e.g., the value of η x (k_cat_/K_m_)_app_ of free enzyme).

2 $$\eta =\frac{3}{\phi }\left[\frac{1}{\tanh\phi }-\frac{1}{\phi }\right]$$

### Stability and reusability of AcT-PDA composites

Having demonstrated that AcT within the AcT-PDA composite retains a significant degree of its native enzyme activity, we proceeded to evaluate the thermal stability of AcT within the composite coating in comparison to free enzyme. The AcT-PDA composite lost ~ 40% of its activity, and approximately 50% activity after 8 h (Fig. 3c). First order kinetics was used to calculate the deactivation rate (k_d_). This value was determined to be 0.094 h^−1^ (Supplementary Fig. 3). The AcT-PDA composite was highly reusable under classical operating conditions of 25 °C. Following each run of 10 min, the wells containing the AcT-PDA coatings were extensively washed and enzyme activity was measured. As shown in Fig. 3d, full activity was retained even after 10 cycles, indicating that the enzyme was highly stable at operating conditions within the PDA coating.

### Antibacterial activity

**Figure 4:**
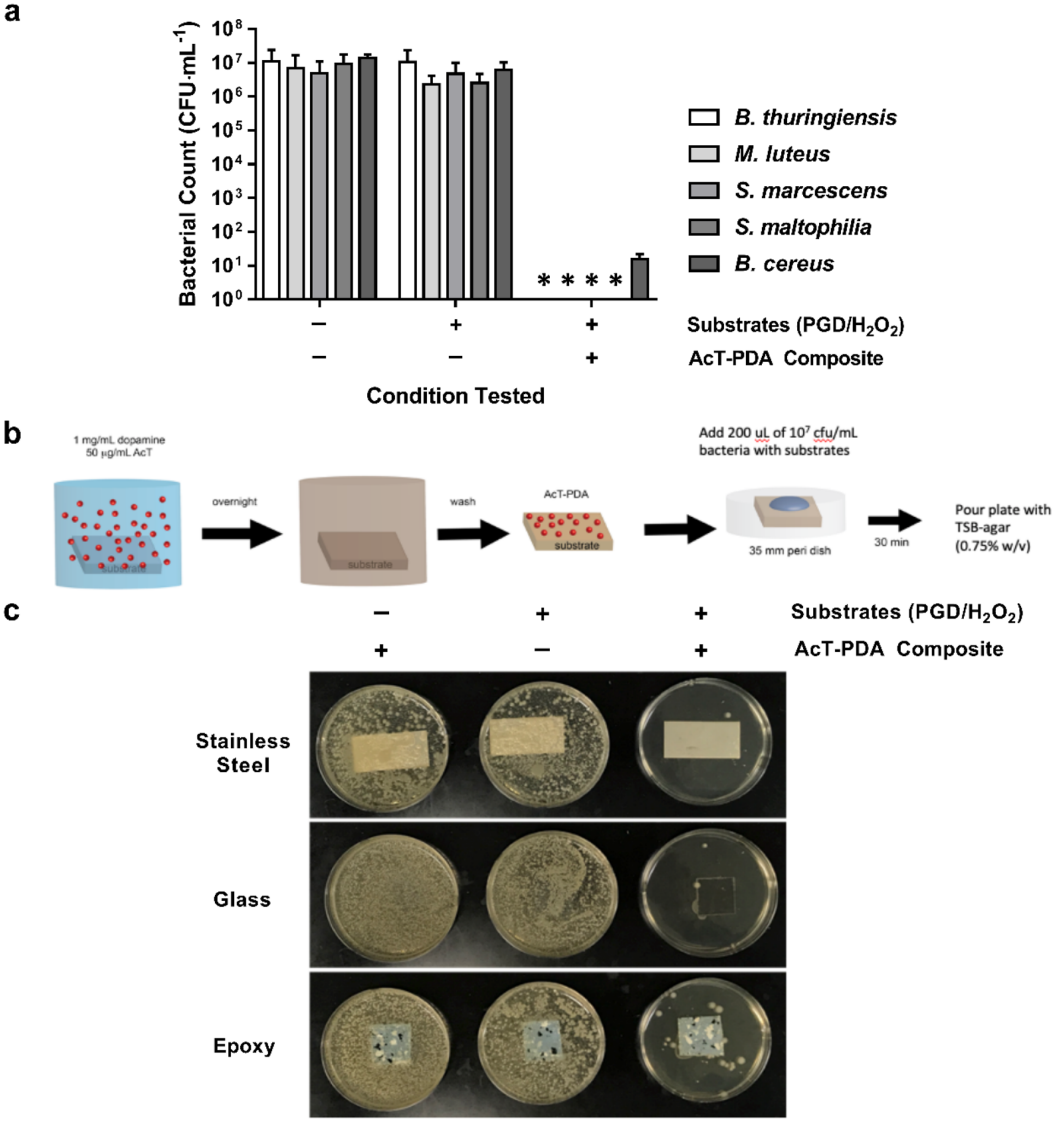
Antimicrobial activity of AcT-PDA against different bacterial strains. (**a**) Broad-spectrum antimicrobial activity observed for AcT-PDA composites (50 μg AcT mg^−1^ PDA) on polystyrene against all bacteria challenged as determined by reduction in CFU through conversion of PGD/H_2_O_2_ into PAA. Four of five bacteria challenged resulted in no CFU being observed and are indicated by asterisks. All tests of coating activity were performed with *n* = 2 replicates and results are shown as mean ± standard deviation. (**b**) Diagram showing method for making AcT-PDA coatings (50 μg AcT mg^−1^ PDA) and demonstrating their self-decontaminating capabilities on various surfaces when presented with a microbial challenge (200 μL at 10^7^ CFU mL^−1^). (**c**) Pictures demonstrating killing effectiveness of AcT-PDA coatings on glass, stainless steel, and epoxy against *B. cereus* using method in (**b**) when microbial challenge was added to bare or AcT-PDA coated surfaces and incubated for 30 min with or without substrates (10 mM PGD and 1 mM H_2_O_2_).

The bactericidal activity of AcT-PDA composite coatings was tested against various bacterial strains at 10^7^ CFU mL^−1^ cell challenges. Using *Bacillus cereus* as a model bacterium, 30 min incubation of the AcT-PDA composite coating in the presence of 10 mM PGD and 1 mM H_2_O_2_ (referred to as PGD/H_2_O_2_ in Fig. 4 without the AcT-PDA composite) resulted in a 6-log kill (Fig. 4a); it should be noted that higher concentrations of H_2_O_2_, i.e., 10 mM used in the activity assays, led to peroxide-induced cell killing in the absence of enzyme (data not shown). The absence of enzyme (PGD/H_2_O_2_ and PBS alone) led to no observable bactericidal activity. Moreover, complete cell killing was obtained even after five reuses (data not shown). We then expanded to additional bacteria, including the Gram-positive *Bacillus thuringiensis* and *Micrococcus luteus* and the Gram-negative opportunistic pathogens *Serratia marcescens* and *Stenotrophomonas maltophilia*, as a representative cross-section of bacteria. In all cases, apart from *B. cereus*, approximately 7-log killing was observed with no remaining live cells after 30 min (Fig. 4a). Thus, the AcT-PDA composite coatings yielded very high broad-spectrum antibacterial activity.

### Antimicrobial activity on various surfaces

To demonstrate the potential of the AcT-PDA composite as a self-decontaminating coating, we applied it onto several surfaces, including epoxy, stainless steel, and glass coupons. These surfaces were extensively washed to remove any AcT that leached from the surface, then challenged with 200 μL of a 10^7^ CFU mL^−1^ suspension of *B. cereus* containing 10 mM PGD and 1 mM H_2_O_2_, and incubated for 30 min at room temperature (Fig. 4b). After 30 min, an agar overlay was added, the petri dishes incubated for 24 h and the resulting colonies were visualized. As shown in Fig. 4c, AcT-PDA coatings possessed high antimicrobial activity on all surfaces in the presence of PGD and H_2_O_2_, with no colonies observed near the coupon for AcT-PDA coatings on stainless steel and glass, and a minimal number of colonies on epoxy. In the absence of the AcT-PDA coating and in the PGD/H_2_O_2_ (control), there was clear growth of colonies.

### Rapid antiviral activity of AcT-PDA composites

**Figure 5:**
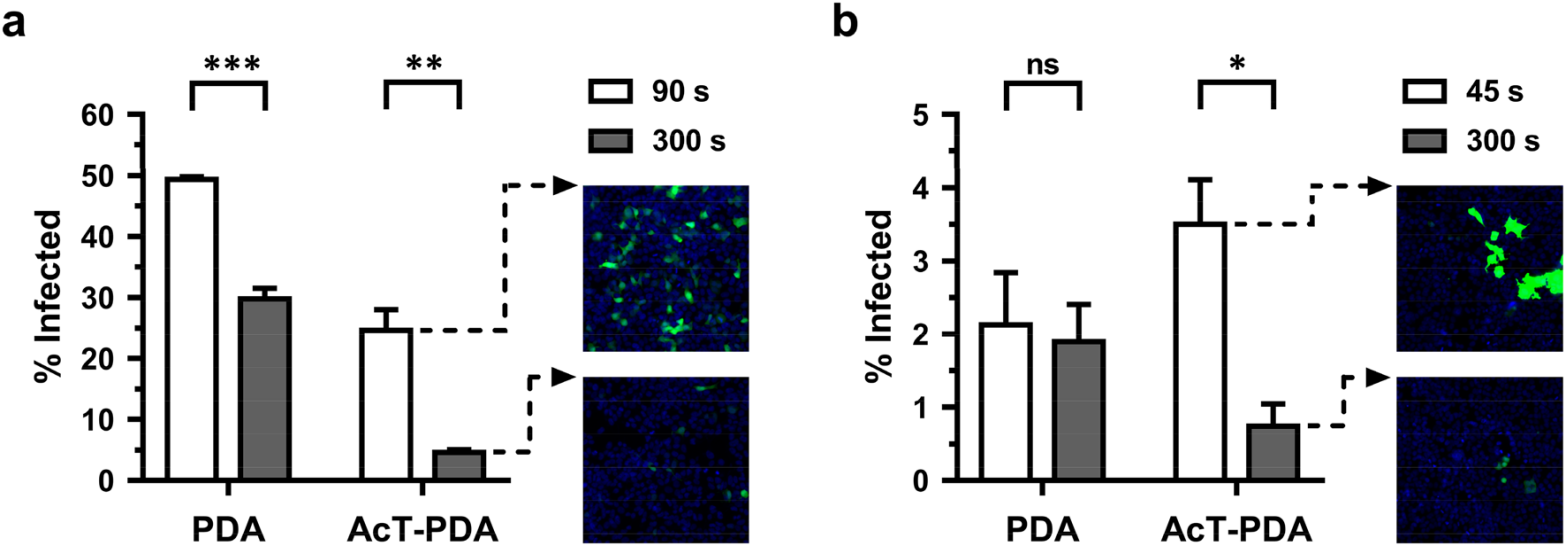
Antiviral activity of AcT (10 μg mL^−1^)-PDA composite against different viruses. (**a**) VSVg pseudotyped lentivirus and (**b**) SARS-CoV-2 pseudovirus in 300 s at 25 °C. The x-axis shows the condition tested and the y-axis shows the % infected. The black bars represent *t* = 90 s and *t* = 45 s for the lentivirus and pseudovirus, respectively, while the gray bars represent *t* = 300 s. The representative images show the virus-infected cells after treatment with AcT-PDA at initial time point (t = 90 s for lentivirus and t = 45 s for pseudovirus) and at 300 s. Nuclei are stained blue with Hoechst 33342. A student’s t-test was used to calculate statistical significance. Error bars represent mean ± SEM across three replicates. *p < 0.05, **p < 0.01, ns-not significant.

Rapid deactivation of virus particles on contaminated surfaces is critical to achieve reduced viral load and transmission. To this end, we examined two viruses, including a VSV-g pseudotyped lentivirus and a SARS-CoV-2 spike pseudotyped lentivirus, both carrying an *Egfp* reporter gene (Supplementary Fig. 4). The AcT-PDA composite coatings at 10 µg mL^−1^ AcT, 5 mM PGD, and 2.5 mM H_2_O_2_ were capable of eliminating > 80% of functional viral titer for a lentivirus (using HEK293T as susceptible target cells) and > 70% of functional viral titer for a SARS-CoV-2 pseudovirus (using HEK293T-Ace2 as susceptible target cells) upon exposure to the composites and the substrates in just 5 min, which is consistent with rapid decontamination (Fig. 5). While the PDA plus substrates condition (no AcT) revealed a drop in viral infectivity for VSV-g lentivirus (possibly due to the H_2_O_2_), the magnitude of this drop was much higher in the presence of the enzyme due to the generation of PAA. Another way to assess the effectiveness of the AcT-PDA composite is to calculate the ratio of infectivity between the two time points. For the VSV-g lentivirus, the ratio of functional virus particles of PDA plus substrates at 5 min and 1.5 min is ~ 0.6, while the same ratio for AcT-PDA plus substrates is ~ 0.2. Similarly, for SARS-CoV-2, the ratio of functional virus particles of PDA plus substrates at 5 min and 0.75 min is ~ 1.0, while the same ratio for AcT-PDA plus substrates is ~ 0.2. Thus, the AcT-PDA composite was effective in neutralizing both VSV-g and SARS-CoV-2 pseudovirus particles in a very short time, which mimics simple surface contact.

In addition, there was a difference in the % infected value between the two virus strains at the first time point (*t* = 1.5 min for lentivirus and *t* = 0.75 min for pseudovirus). This was most likely due to the differences in the MOI of the virus used in the antiviral experiment. SARS-COV-2 pseudovirus produced much lower final viral titers (~ 100-fold) than VSV-g pseudotyped lentivirus. Hence, a lower MOI was used for the SARS-COV-2 deactivation experiment. The low viral titers for the SARS-COV-2 pseudovirus is to be expected since the addition of spike glycoprotein in a normal coronavirus occurs at the Endoplasmic Reticulum-Golgi intermediate compartment (ERGIC)^49^. The pseudovirus titer obtained can be much lower if the envelope glycoproteins are acquired at organelles like Golgi or ER instead of at the plasma membrane^50^. Indeed, deletion of 19 amino acids on the C-terminus of the spike protein that is responsible for transporting the spike protein to the ER has been shown to result in higher pseudoviral titer as the spike protein is no longer retained in the ER but translocates to the plasma membrane^51^.

## Conclusions

In summary, we have demonstrated that deposition of an AcT-PDA composite coating, prepared in a single-step by adding AcT to a PDA solution and then drying on a surface, provided a versatile platform for enzyme immobilization and resulted in highly active antibacterial and antiviral surfaces. The incorporated AcT retained ~ 40% of its native solution catalytic turnover. The coatings were also thermostable, with approximately 50% activity loss in 8 h at 70 °C. The enzyme-containing coating was highly effective against significant challenges of a diverse range of both Gram-positive and Gram-negative bacteria, as well as two lentivirus strains, including one genetically modified to serve as a SARS-CoV-2 pseudovirus. In addition to simple drop-cast coatings, antibacterial activity was obtained with AcT-PDA bound to plastic, metal, and glass surfaces. Our study provides a scalable route to the creation of biocatalytic coatings by a simple single-step immobilization procedure. Such coatings may be useful in myriad environments, including social infrastructure (e.g., schools and transportation hubs), healthcare facilities, food processing facilities, and biomanufacturing plants.

## Supplementary Information

Supplementary Information.
