# Supplementary material for: Facile fabrication of antibacterial and antiviral perhydrolase-polydopamine composite coatings
Source: Sci Rep. 2021 Jun 14;11:12410. doi: 10.1038/s41598-021-91925-6 (PMC8203652; doi:10.1038/s41598-021-91925-6)
Supplement: Supplementary file 1 — Supplementary Information. [file 41598_2021_91925_MOESM1_ESM.docx]

**Supplementary Information for**

**Facile Fabrication of Antibacterial and Antiviral Perhydrolase-Polydopamine Composite Coatings**

Li-Sheng Wang^1≠^, Shirley Xu^1≠^, Sneha Gopal^1^, Eunsol Kim^1^, Domyoung Kim^1^, Matthew Brier^1^,

Kusum Solanki^1^ and Jonathan S. Dordick^1,2^*

^1^Department of Chemical and Biological Engineering and Center for Biotechnology & Interdisciplinary Studies, Rensselaer Polytechnic Institute, 110 8^th^ Street, Troy, New York 12180, USA

^2^Department of Biological Sciences and Department of Biomedical Engineering

Rensselaer Polytechnic Institute, 110 8^th^ Street, Troy, New York 12180, USA

**
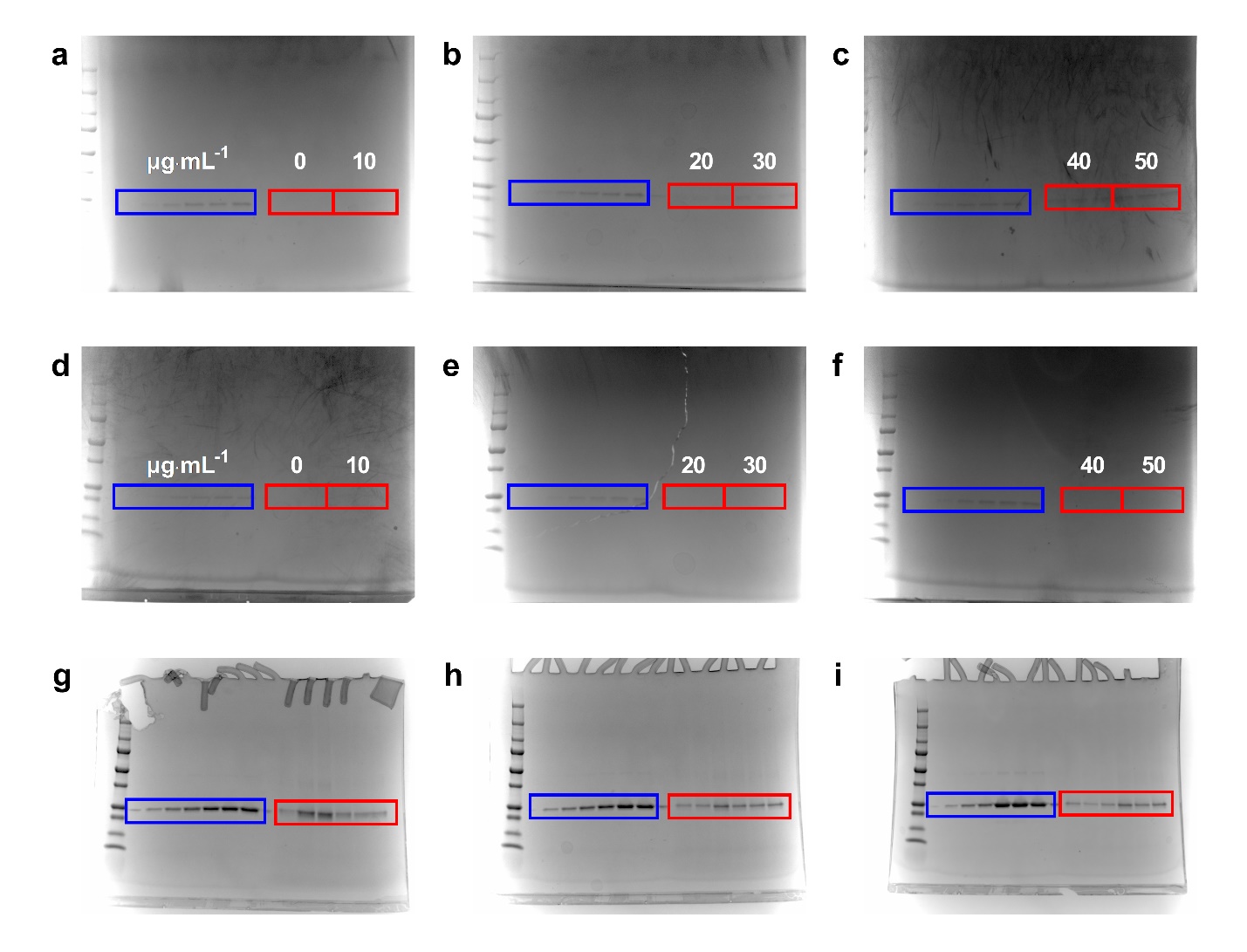
**

**Supplementary Figure 1. Original full images of SDS-PAGE.** Following the overnight incubation of 0.1 mg dopamine and 0-10 μg AcT in 100 μL PBS solution, the remaining unreacted dopamine/AcT solutions were collected and run on SDS-PAGE. (**a-c**) Images of gels for residual AcT in dopamine/AcT solutions for two-step coating method using (**a**) 0 and 10, (**b**) 20 and 30, and (**c**) 40 and 50 µg AcT·mL^-1^ reaction. (**d-f**) Images of gels for residual AcT in dopamine/AcT solutions for one-step coating method using (**d**) 0 and 10, (**e**) 20 and 30, and (**f**) 40 and 50 µg AcT·mL^-1^ reaction. (**g-i**) Images of gels for residual AcT in dopamine/AcT solutions for two-step coating method using 50-100 µg AcT·mL^-1^ reaction. For each gel shown in (**a-i**) one replicate of each concentration (0-50 µg·mL^-1^ for (**a-f**) and 0-100 µg·mL^-1^ for (**g-i**)) of the AcT standards was included (blue box) to allow consistent normalization and quantification. For each gel shown in (**a-f**), the labelled red boxes indicate the positions where the residual AcT from replicate preparations (*n* = 3) were measured. For each gel shown in (**g-i**), the red boxes indicate the positions where the residual AcT from a single preparation at 50-100 µg AcT·mL^-1^ reaction were measured, with all three gels combining to showing the replicates (*n* = 3) for each of the aforementioned one-step AcT-PDA preparations.

**
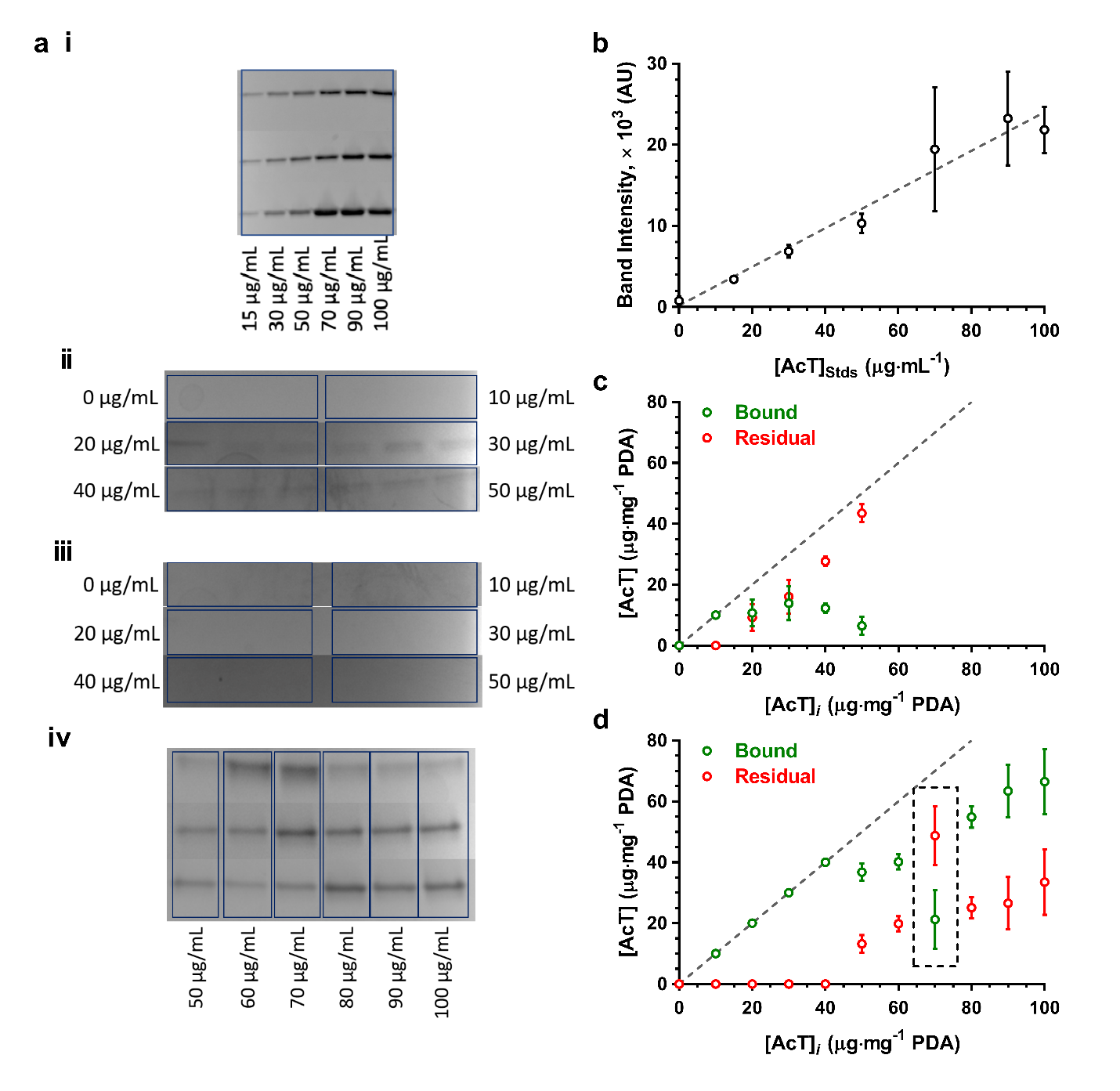
****Supplementary Figure 2. Quantification of immobilized AcT using SDS-PAGE.** Following the overnight incubation of 0.1 mg dopamine and 0-10 μg AcT in 100 μL PBS solution, the remaining unreacted dopamine/AcT solutions were collected and run on SDS-PAGE. (**a**) Cropped and modified (to better visualize bands) gel images from Supplementary Fig. 1 showing band intensity for (**i**) AcT standards (from blue boxes in Supplementary Fig. 1g-i), (**ii**) two-step coating method using 0-50 μg∙mg^-1^ PDA (from red boxes in Supplementary Fig. 1a-c), (**iii**) one-step coating method using 0-50 μg AcT∙mg^-1^ PDA (from red boxes in Supplementary Fig. 1d-f), and (**iv**) one-step coating method using 50-100 μg AcT∙mg^-1^ PDA (from red boxes in Supplementary Fig. 1g-i). At low loadings (< 50 μg AcT∙mg^-1^ PDA), the concentration of unbound AcT was too low to be detected by SDS-PAGE. (**b**) Example standard curve of band intensity as a function of pure AcT concentration obtained by image analysis of (**a, i**) SDS-PAGE using ImageJ. Calculated concentration of bound AcT (green) in coating using SDS-PAGE measured unbound (residual, red) AcT in solution from (**c**) two-step coating method shown in (**a, ii**) and (**d**) one-step coating method shown in (**a, iii-iv**). Gray dashed lines in (**c**) and (**d**) show maximum possible AcT loading in AcT-PDA coatings based on initial AcT concentration, [AcT]*_i_*, at start of synthesis. The apparent poor loading of AcT into AcT-PDA coatings for the one-step method using 70 μg AcT∙mg^-1^ PDA (denoted by blacked dashed rectangle), as measured by unusual levels of AcT remaining in solution, was unknown, and for this reason this loading was not used during further studies shown in this work. All tests of AcT loading using each method were performed with *n* = 3 replicates, with 50 μg AcT∙mg^-1^ PDA condition overlapping between one-step coating method tests (total *n* = 6). All data shown as mean ± standard deviation; error bars not plotted for some points that have very small standard deviations.


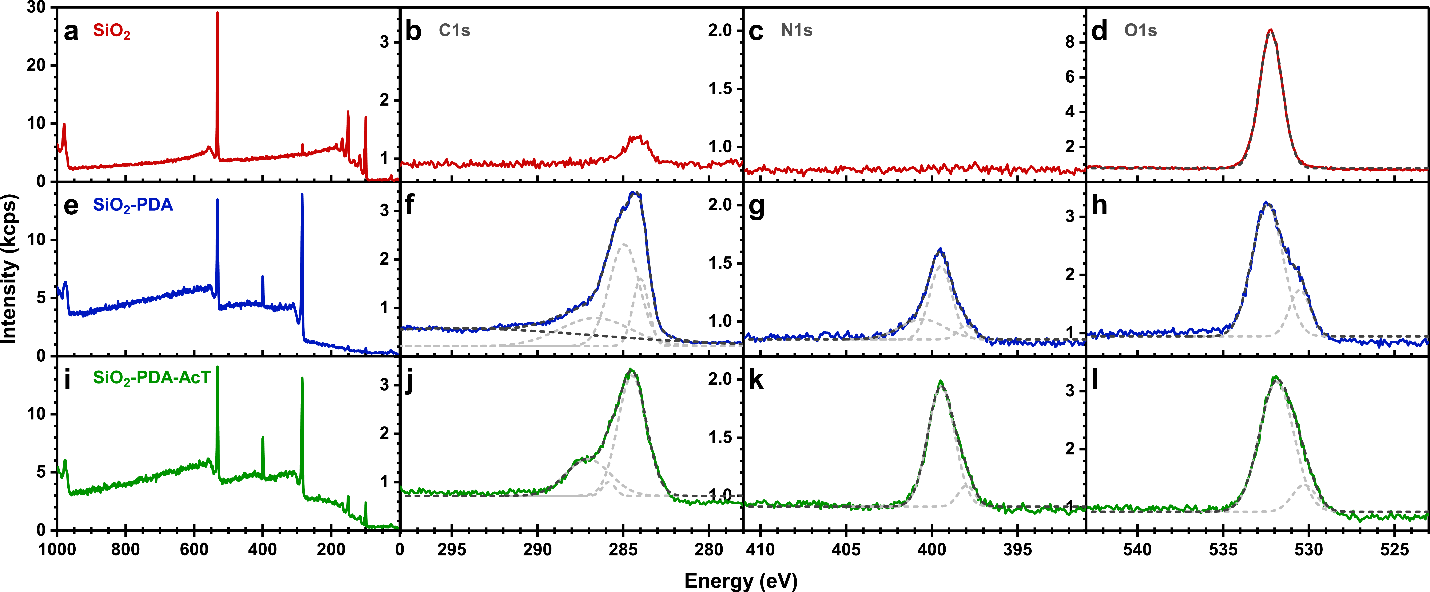


**Supplementary Figure 3. X-ray photoelectron spectroscopy (XPS) analysis of AcT-PDA composite coatings on silicon wafers.** Red curves show base silicon dioxide (SiO­_2_) layer on silicon wafer through (**a**) overall XPS spectrum, (**b**) minor C1s peak due to any surface particulate, (**c**) no N1s peak, and (**d**) O1s peak from SiO_2_. Blue curves show pure PDA coating on silicon wafer (SiO­_2_-PDA) through (**e**) overall XPS spectrum, (**f**) C1s peak from PDA layer, (**g**) N1s peak from PDA layer, and (**h**) O1s peaks from PDA and SiO_2_. Green curves show one-step AcT-PDA coating on silicon wafer (SiO­_2_-PDA-AcT) through (**i**) overall XPS spectrum, (**j**) C1s peaks from PDA and AcT, (**k**) N1s peak from PDA and AcT, and (**l**) O1s peak from PDA and AcT. Black dashed lines in (**d**), (**f-h**), and (**j-l**) show overall peak fitting while gray dashed lines show Gaussian deconvolution into possible individual peaks showing different binding states of C, N, and O within the respective materials.

**
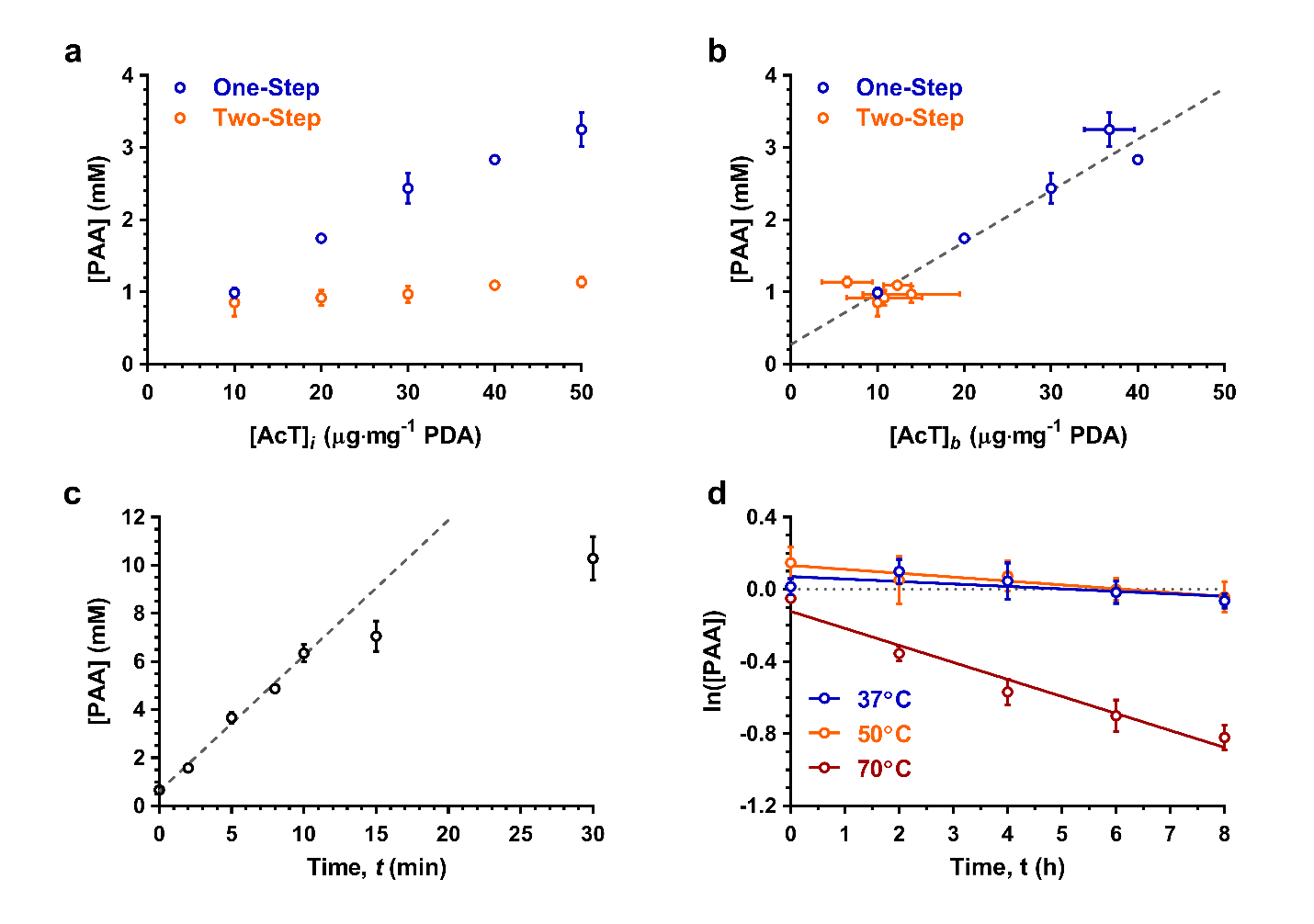
**

**Supplementary Figure 4. Preliminary activity studies of AcT-PDA coatings synthesized using both one-step and two-step methods.** Coatings synthesized using each method were incubated with a substrate solution of 100 mM PGD and 10 mM H_2_O_2_. After incubation, activity was measured by determining PAA generated using the ABTS assay. (**a**) Comparison of PAA generated by AcT-PDA coatings as a function of initial AcT concentration, [AcT]*_i_*, using 0-50 μg AcT∙mg^-1^ PDA over 30 min incubation period with PGD/H_2_O_2_ solution. (**b**) Modified comparison PAA generation shown in (**a**) based on actual AcT concentration in bound in AcT-PDA coatings, [AcT]*_b_*, as determined by SDS-PAGE and shown in Supplementary Fig. 2c and d for the two- and one-step methods, respectively. Gray dashed line shows linear trend in concentration of PAA, [PAA], as function of [AcT]*_b_*. (**c**) PAA production as a function time to identify the linear region (gray dashed line) of AcT activity for AcT-PDA coatings loaded using 50 μg AcT∙mg^-1^ PDA. Findings determined all activity assays for comparisons of AcT-PDA coating activity would use 10 min incubations. (**d**) Confirmation of first order decay in activity of AcT-PDA coatings loaded using 50 μg AcT∙mg^-1^ PDA over time for each temperature condition tested in Fig. 3d. Linear regression analysis of mean values confirmed significant (*p* < 0.05 as compared to slope of 0) first order decay for 50 and 70°C with slopes of −0.021 ± 0.004 and −0.094 ± 0.011, respectively, compared to the non-significant decay of 37C with a slope of −0.014 ± 0.008. As visualized on the plot in (**d**), the more rapid decay at 70°C is clearly represented by the more negative slope as compared to the 37 and 50°C conditions. All tests of coating activity were performed with *n* = 3 replicates. All data shown as mean ± standard deviation; error bars not plotted for some points that have very small standard deviations.

a


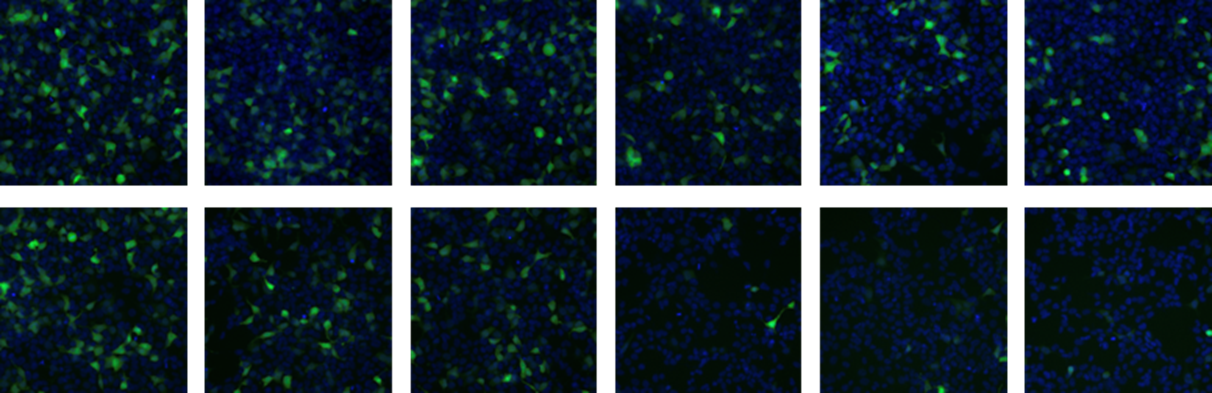


90 sec

300 sec

PDA + 2.5 mM PGD + 5 mM H_2_O_2_

10 µg/mL AcT-PDA + 2.5 mM PGD + 5 mM H_2_O_2_


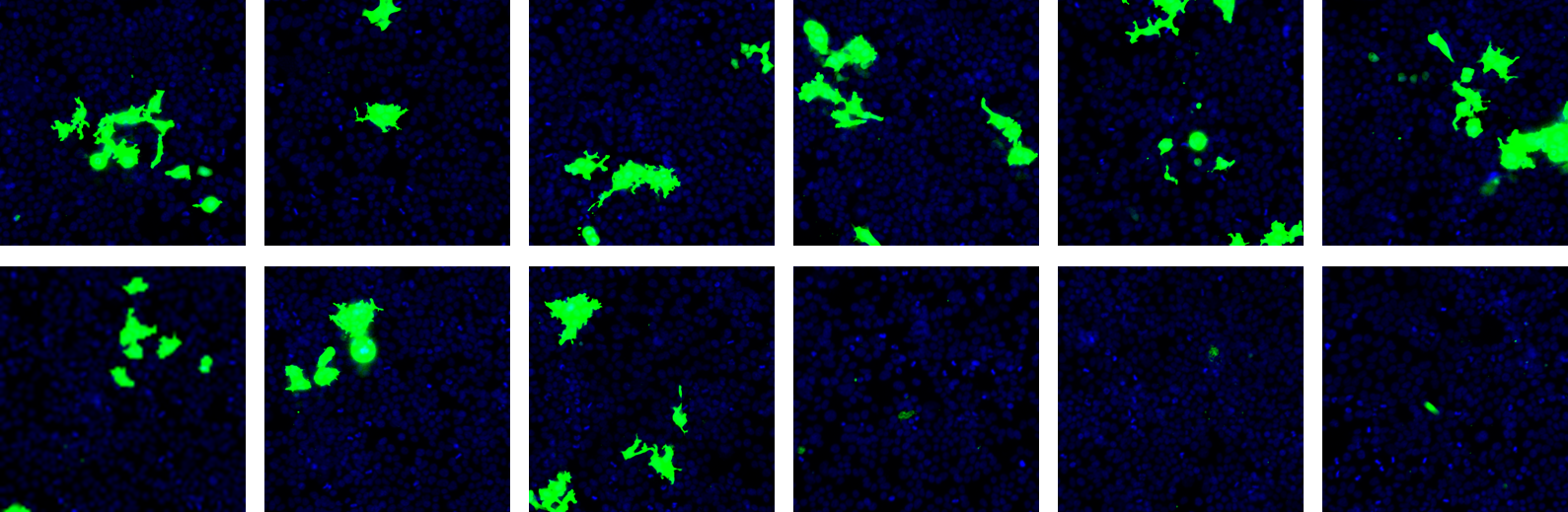


45 sec

300 sec

PDA + 2.5 mM PGD + 5 mM H_2_O_2_

10 µg/mL AcT-PDA + 2.5 mM PGD + 5 mM H_2_O_2_

b

**Supplementary Figure 5.** **Fluorescent images of virus-infected HEK293T cells demonstrating rapid reduction in viral titer by AcT-PDA coatings.** Representative fluorescent images showing viral infection (GFP expression, green) of HEK293T cells (Hoechst-stained nuclei, blue) with (**a**) VSV-G lentivirus and (**b**) SARS-COV-2 pseudovirus using a number of treatment conditions. All conditions tested with *n* = 3 replicates.
